# Type distribution of human papillomavirus among adult women diagnosed with invasive cervical cancer (stage 1b or higher) in New Zealand

**DOI:** 10.1186/1471-2334-14-374

**Published:** 2014-07-08

**Authors:** Peter Sykes, Kusuma Gopala, Ai Ling Tan, Diane Kenwright, Simone Petrich, Anco Molijn, Jing Chen

**Affiliations:** 1 University of Otago Christchurch, Christchurch Women’s Hospital, Otago, New Zealand; 2 GlaxoSmithKline Vaccines, Bangalore, India; 3 National Women’s Hospital, Auckland District Health Board City, Auckland, New Zealand; 4 University of Otago, Wellington, Capital Coast District Health Board, Otago, New Zealand; 5 Dunedin Hospital Southern District Health Board, Dunedin, New Zealand; 6 DDL Diagnostic Laboratory, Rijswijk, The Netherlands; 7 GlaxoSmithKline Vaccines, 150 Beach Road, Gateway West #22-00, 189720 Singapore, Singapore

**Keywords:** Epidemiology, Human papillomavirus, Invasive cervical cancer, New Zealand

## Abstract

**Background:**

Human papillomavirus (HPV) is the necessary cause of cervical cancer. Published data on the epidemiology of HPV in women with invasive cervical cancer (ICC) in New Zealand (NZ) are limited. This cross-sectional study investigated the distribution of high-risk and low-risk HPV types in cervical specimens collected from women throughout NZ who had been diagnosed with ICC between 2004 and 2010.

**Methods:**

Women aged ≥18 years, with ICC International Federation of Gynecology and Obstetrics stage Ib or greater were identified from the five tertiary public hospitals in NZ regularly treating women with ICC. Women were enrolled in the study only after obtaining informed consent. Stored, formalin fixed, paraffin-embedded cervical specimens were retrieved and histopathologically reviewed to confirm the diagnosis of ICC. Cervical specimens were tested for HPV using polymerase chain reaction-short fragment10; HPV DNA was detected using DNA enzyme immunoassay and typed by reverse hybridization line probe assay.

**Results:**

242 women were enrolled and ICC was histologically confirmed in 227 samples. HPV infection was detected in 88.5% (n = 201; 95% CI: 83.7–92.4) of women with ICC; high-risk HPV types were detected in 87.2% of women. The most commonly detected HPV types were HPV-16 (51.1%) and HPV-18 (20.7%), followed by HPV-31 (4.0%), HPV-45 and HPV-52 (3.1% each). Overall, HPV distribution was highest (94.3%) in women aged 30–39 years at diagnosis and a higher distribution of HPV-16 (68.8%) was observed in women younger than 30 years. The overall distribution of HPV types between Maori and non-Maori women were similar. HPV-positive women with ICC stage II or greater were less likely to be infected with HPV-16/18 (*P* = 0.002) or HPV-18 (*P* = 0.029) compared with the other high-risk types. Single type infection and multiple infections were detected in 93.5% and 5.5% of women, respectively.

**Conclusions:**

HPV-16, HPV-18, HPV-31, HPV-45 and HPV-52 were the most commonly detected high-risk HPV types. Findings from the study fill an important data gap on HPV type distribution from NZ which will help facilitate better understanding of the epidemiology of HPV in NZ women.

**Clinical trial registration:**

NCT01328028.

## Background

Cervical cancer (CC) is the third most common cancer in women worldwide with the burden being highest in developing countries [1]. The incidence of CC in New Zealand (NZ) and Australia is among the lowest in the world [2], however CC remains an important, largely preventable cause of morbidity and mortality and is 3rd most common among younger women aged 15–44 years [3]. NZ has a population of around 2.18 million women [3] and the NZ Cancer Registry estimated that there were 141 new CC cases in 2009, 179 in 2010 and 161 in 2011 [4].

The low CC incidence in NZ is in part explained by the existence of the National Cervical Screening Programme (NCSP). Established in 1990, the NCSP recommends cervical smear tests once every three years for women aged 20–70 years and has been associated with a marked reduction in the mortality and incidence of CC [5]. Indigenous populations of NZ, such as Maori women, have historically had low uptake rates in the NCSP. In 2006, the NCSP screening coverage was 46.6% for Maori women compared with 75.7% for non-Maori non-Pacific women [6]. While much is being done to reduce the inequalities in healthcare access, CC incidence and mortality disproportionately affect Maori women [6,7]. In 2009, the age-standardized rate of CC was 10.4 per 100,000 in Maori women compared with 4.8 per 100,000 in non-Maori women [8].

It is well established that persistent infection with high-risk (HR) human papillomavirus (HPV) is the most important cause of CC, causing the vast majority of cases [9]. Fifteen HPV types have been epidemiologically established as oncogenic or HR: 16, 18, 31, 33, 35, 39, 45, 51, 52, 56, 58, 59, 68, 73 and 82. A further twelve types have been classified as low-risk (LR): 6, 11, 40, 42, 43, 44, 54, 61, 70, 72, 81, and CP6108 [10]. HR-HPV types 16 and 18 account for around 70% of all CC cases [11].

As CC is one of the most preventable types of cancer, even the low incidence rates of CC in NZ are a cause of concern and these have the potential to be further lowered by comprehensive vaccination initiatives [12,13]. Two HPV vaccines are currently licensed and available in NZ: the quadrivalent HPV 6/11/16/18 vaccine (*Gardasil*®, Merck, US), and the bivalent HPV 16/18 vaccine (*Cervarix*®, GlaxoSmithKline, Belgium) [14,15]. The NZ Ministry of Health initiated a mass HPV vaccination programme in 2008, initially for girls from the 1990–1991 birth cohorts. This was subsequently expanded and HPV vaccination became part of the National Immunization Schedule for girls aged 12 years in 2009 [15].

Data addressing the epidemiology of HPV among women with invasive cervical cancer (ICC) in NZ are limited. As the oncogenicity of specific HPV variants appears to vary geographically and depending on the ethnic origin of the population studied [16]. To understand the potential impact of national HPV vaccine programs on cervical cancer incidence baseline epidemiological data are required.

This study aimed to evaluate the prevalence of HPV types 16 and 18 among women diagnosed with ICC in NZ, the prevalence of HR and LR HPV types, and co-infection of HPV-16 and HPV-18 with other HR-HPV types.

## Methods

### Study design and population

This cross-sectional, hospital-based, epidemiological study was conducted between April 2009 and April 2011 in the five tertiary hospitals in NZ regularly treating ICC: National Women’s Hospital (Auckland), Dunedin Hospital (Dunedin), Christchurch Hospital (Christchurch), Wellington Hospital (Wellington; including cases from Palmerston North) and Waikato Hospital (Waikato).

Hospital records between 2004 and 2010 were used to identify women aged 18 years and above with a diagnosis of ICC International Federation of Gynecology and Obstetrics (FIGO) stage Ib or greater [17]. ICC included all squamous cell carcinoma (SCC), adenocarcinoma (ADC) and adenosquamous carcinoma (ASC) types while neuroendocrine small cell carcinoma and sarcoma were excluded. Consent from the patient, or in the case of deceased patients the next of kin, to access tissue and clinical notes was obtained. Formalin fixed and paraffin-embedded cervical tissue samples were then procured from hospital archives and anonymised. Samples were excluded if they were too thin (<2 mm), or too small (<0.5 cm across), or collected from women after chemotherapy/radiotherapy. Hospital records were also used to capture information in the case report form regarding the demographic characteristics (age, ethnicity), histological diagnosis, year of tissue sample collection and the stage of cancer.

### Laboratory procedures

Cervical tissue samples were sectioned using a sandwich technique and ICC was confirmed in the tissue block by histopathological review at DDL Diagnostic Laboratory (Rijswijk, Netherlands). Patients were excluded if ICC could not be confirmed in the available tissue. HPV DNA was extracted from histopathologically confirmed ICC tissue samples using proteinase K and amplified using the HPV short polymer fragment-10 (SPF10), version 1 polymerase chain reaction (Labo Bio-medical Products, Rijswijk, The Netherlands). Amplified DNA was detected with DNA enzyme immunoassay (DEIA) and typed by reverse hybridization line probe assay (LiPA, version 1) using 25 type-specific hybridization probes which detected 14 HR (HPV-16, 18, 31, 33, 35, 39, 45, 51, 52, 56, 58, 59, 66 and 68) and 11 LR-HPV types (HPV-6, 11, 34, 40, 42, 43, 44, 53, 54, 70 and 74) [18,19]. Positive and negative controls were used for each run to monitor DNA isolation, PCR amplification, HPV detection and genotyping. Re-testing of HPV negative samples was conducted after diluting DNA tenfold.

### Statistical analyses

Based on the global prevalence rates of HPV types in ICC [20] and allowing for ~ 30% non-evaluable samples, target enrolment was 285 women to provide 200 samples evaluable for the prevalence of HPV-16 and HPV-18 with a precision of ± 7% and ± 5%, respectively.

All analyses were descriptive and the percentage of women was tabulated with their corresponding exact 95% confidence interval (CI). Exploratory logistic regression analysis was performed to estimate the effect of age at sample collection, ethnic origin (Maori/non-Maori) and stage of cancer (stage Ib/stage II and above) on HPV detection rate in the histologically confirmed cervical tissue samples, and also on the prevalence of HPV-16, HPV-18 and HPV-16/18 in the HPV positive cervical tissue samples. All statistical analyses were performed using SAS® software version 9.2. (SAS Institute Inc., Cary, NC, USA).

### Ethical considerations

The study was approved by the Health and Disability Ethics Committees-Multi-Region Ethics Committee, Wellington, NZ and was conducted in accordance with the Declaration of Helsinki and Good Clinical Practice guidelines.

## Results

### Study population and histopathology

**Figure 1:**
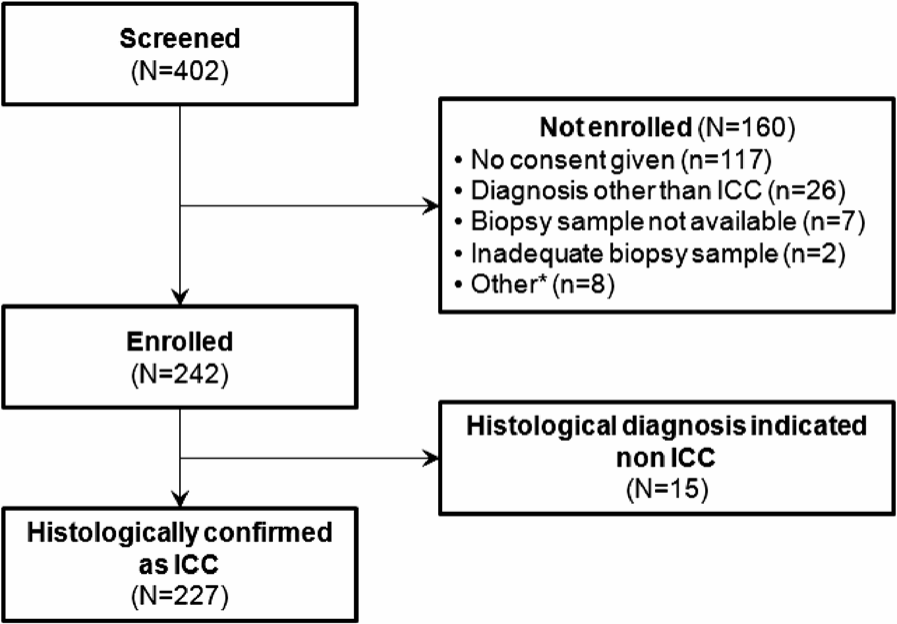
**Subject screening and enrolment.** *Reasons included: subjects contacted by other study site (n = 6); uncertain diagnosis (n = 1) and not specified (n = 1). ICC, invasive cervical cancer.

Subject screening and enrolment is summarized in Figure 1. A total of 242 women were enrolled and the diagnosis of ICC was histologically confirmed in 227 women. The mean age (standard deviation [SD]) of women at the time of diagnosis in the histologically confirmed group was 50.7 (±16.7) years. Women of Maori ethnicity accounted for 15.0% (34/227) of the ICC women with the remaining 85.0% (193/227) of non-Maori origin.

Of the 227 confirmed ICC cases, 159 (70.0%) were SCC, 61 (26.9%) ADC and 7 (3.1%) ASC. A total of 115 out of 227 women (50.1%) were assessed as having FIGO stage Ib cancer while the remaining 112 were diagnosed with cancer of FIGO stage II and above.

### Overall HPV detection rate and type distribution

The overall detection rate and type distribution of HPV are shown in Table 1. HPV DNA was detected in 88.5% (201/227) of histologically confirmed ICC tissue samples, with 93.1% (148/159) SCC and 77.9% (53/68) of ADC/ASC positive for HPV. Among HPV positive samples, 81.1% (163/201) were infected with either HPV 16 or 18. The most common HR HPV types detected among confirmed ICC cases were HPV-16 (51.1%) and HPV-18 (20.7%). HPV-16 was the most prevalent HPV DNA type across the ICC histological subtypes, but HPV-18 was detected in proportionally more ADC/ASC than SCC (35.3% vs. 14.5%). For the LR HPV types detected, HPV-11 and HPV-70 were detected in one cervical sample each and were both of the SCC subtype.

**Table 1:** **HPV detection and type distribution in histologically confirmed ICC tissue samples from New Zealand women (N**^**a**^ **= 227)**

**Histological diagnosis/HPV type**	**All ICC types (N = 227)**		**ICC subtypes**			
			**ADC/ASC (N = 68)**		**SCC (N = 159)**	
	**n**^**b**^	**%**^**c **^**(95% CI)**	**n**	**% (95% CI)**^**d**^	**n**	**% (95% CI)**
**HPV positive**	201	88.5 (83.7–92.4)	53	77.9 (66.2–87.1)	148	93.1 (88.0–96.5)
**Any high-risk HPV**	198	87.2 (82.2–91.3)	53	77.9 (66.2–87.1)	145	91.2 (85.7–95.1)
HPV-16	116	51.1 (44.4–57.8)	27	39.7 (28.0–52.3)	89	56.0 (47.9–63.8)
HPV-18	47	20.7 (15.6–26.6)	24	35.3 (24.1–47.8)	23	14.5 (9.4–20.9)
HPV-31	9	4.0 (1.8–7.4)	0	0.0 (0.0–5.3)	9	5.7 (2.6–10.5)
HPV-45	7	3.1 (1.2–6.3)	2	2.9 (0.4–10.2)	5	3.1 (1.0–7.2)
HPV-52	7	3.1 (1.2–6.3)	0	0.0 (0.0–5.3)	7	4.4 (1.8–8.9)
HPV-59	5	2.2 (0.7–5.1)	0	0.0 (0.0–5.3)	5	3.1 (1.0–7.2)
HPV-33	4	1.8 (0.5–4.5)	1	1.5 (0.0–7.9)	3	1.9 (0.4–5.4)
Other	17*	7.5 (4.4–11.7)	1	1.5 (0.0–7.9)	16	10.1 (5.9–15.8)
**Any low-risk HPV**	2	0.9 (0.1–3.1)	0	0.0 (0.0–5.3)	2	1.3 (0.2–4.5)

^a^number of women with the corresponding diagnosis.

^b^number of women in each category.

^c^percentage of women in each category.

^d^exact 95% confidence interval.

ICC: Invasive cervical cancer; ADC: Adenocarcinoma; ASC: Adenosquamous carcinoma; SCC: Squamous cell carcinoma.

*Includes 2 HPV types unidentifiable by LiPA.

Other refers to HPV-35 (3 ICC samples), 39 (3), 51 (2), 56 (3), 66 (1), 68 (3) and –X (2 HPV types unidentifiable by LiPA).

### HPV detection rate and type distribution by age

**Figure 2:**
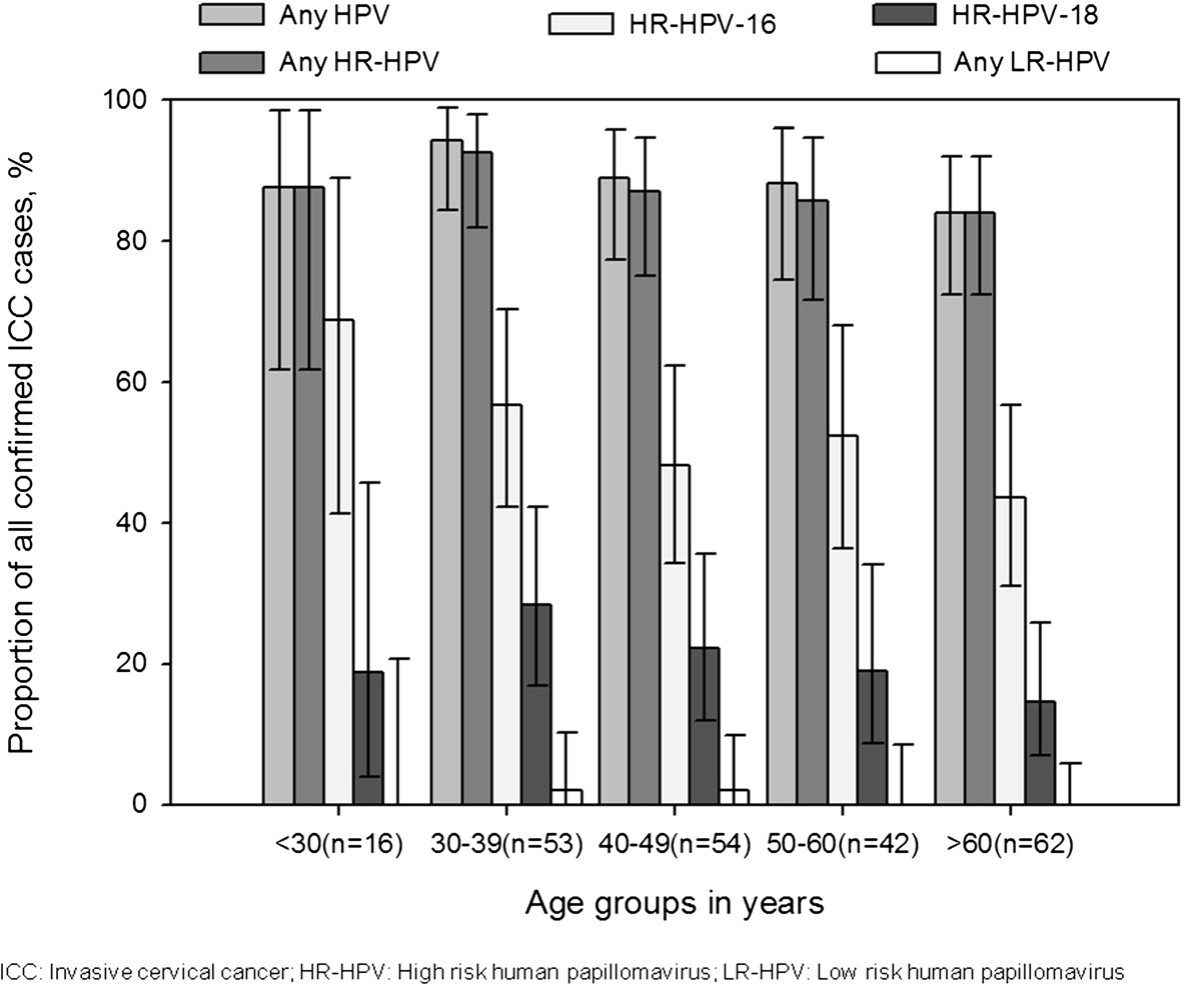
Prevalence of HPV types by age at diagnosis in histologically confirmed ICC tissue samples from New Zealand women (N = 227).

The HPV detection rate and type distribution of HPV by age group is shown in Figure 2. The distribution of any HPV type was highest in women aged 30–39 years at diagnosis. HPV-16 was detected more frequently in women aged less than 30 years compared with those aged greater than 30 years.

### HPV detection rate and type distribution by ethnicity

**Figure 3:**
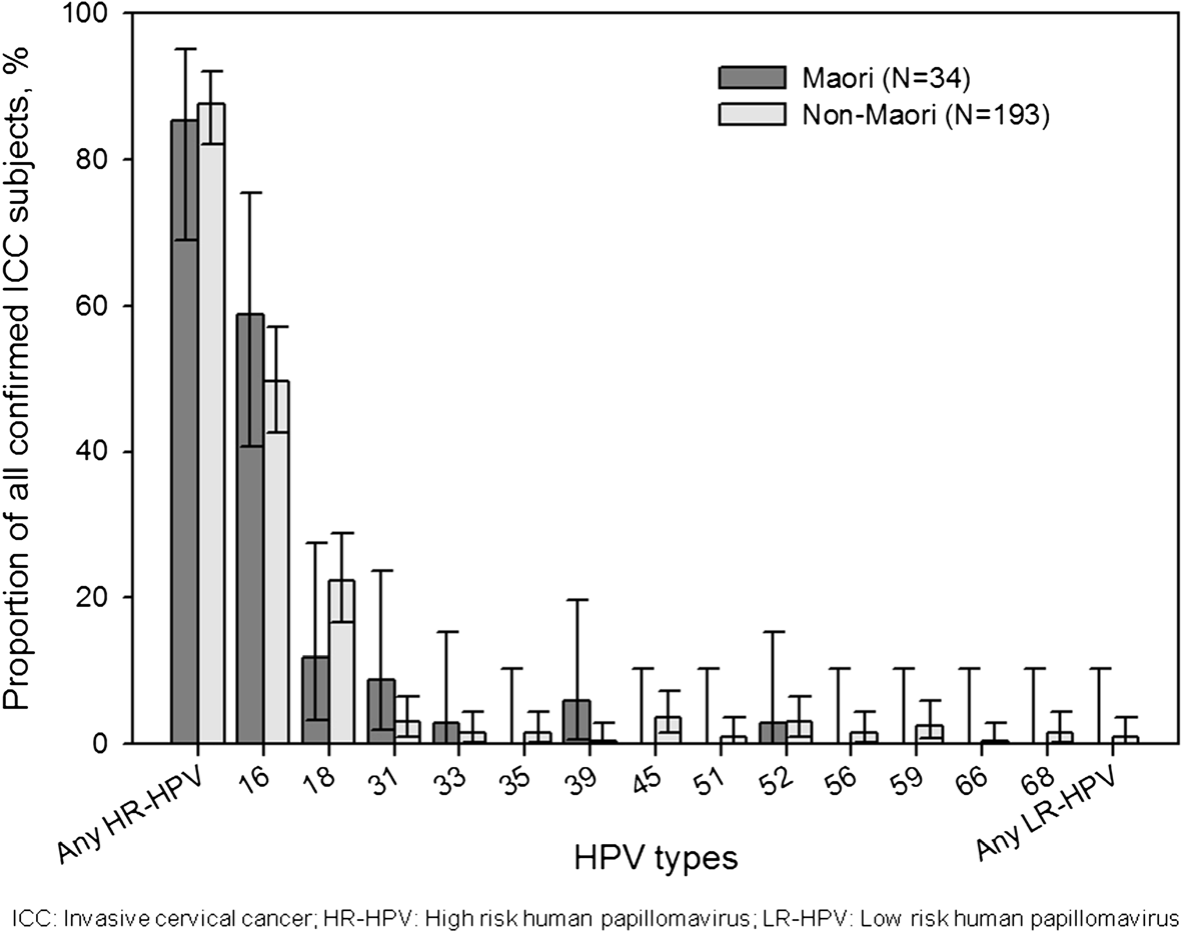
Prevalence of HPV types by ethnic group (Maori or non-Maori) in histologically confirmed tissue samples (N = 227).

HPV detection rate among Maori and non-Maori women is shown in Table 2. The detection rate of HPV in the confirmed ICC samples was similar in the two ethnic groups with 88.2% and 88.6% of cervical tissue samples positive for HPV from Maori and non-Maori women respectively. No significant associations were found between ethnicity and either HPV detection rate, histological subtype or stage of cancer. Additionally, no statistically significant difference was seen in the age at diagnosis between Maori and non-Maori women (Table 2).The prevalence of individual HPV type by ethnicity is shown in Figure 3. Infection with any HR HPV type was similar in Maori (85.3%, 29/34) and non-Maori (87.6%, 169/193) women. HR HPV-16 was present in 58.8% (20/34) Maori women and 49.7% (96/193) non-Maori women. HR HPV-18 was present in 11.8% (4/34) Maori women and 22.3% (43/193) non-Maori women. Minor variations in HPV type distribution were observed in the HPV non-16/-18 types between the two ethnic groups.

**Table 2:** **Comparison of Maori and non-Maori women with histologically confirmed ICC (N**^**a**^ **= 227)**

**Features**		**Maori (N = 34)**	**non-Maori (N = 193)**	***P*-value**^**d**^
		**n**^**b **^**(%**^**c**^**)**	**n (%)**	
By age at diagnosis^*^	Mean (SD^e^)	48.7 (13.5)	51.5 (17.2)	0.507
(years)	Median	48.0	48.0	
	Range	27–72	21–94	
By histological diagnosis^#^	ADC/ASC	9 (26.5)	59 (30.6)	0.630
	SCC	25 (73.5)	134 (69.4)	
By cancer stage^#^	Stage Ib	16 (47.1)	99 (51.3)	0.649
	Stage II and above	18 (52.9)	94 (48.7)	

^a^number of women with the corresponding diagnosis.

^b^number of women in each category.

^c^percentage of women in each category.

^d^Fisher’s Exact test for HPV prevalence in each histological diagnosis; Wilcoxon 2-Sample Test for age at diagnosis, Chi-square test for histological diagnosis and cancer stage.

^e^standard deviation.

^#^Hypothesis tested any association between the features and the ethnic groups.

^*^Hypothesis tested any significant difference with respect to age between the ethnic groups.

### HPV detection rate and type distribution by stage of cancer

Among HPV positive cases (201/227), women with FIGO cancer stage II and above were less likely to be infected with HPV-16/18 compared with women who had FIGO cancer stage Ib when adjusted for ethnicity (adjusted odds ratio 0.3, 95% CI: 0.1–0.6; *P* = 0.002). Similarly, women with FIGO cancer stage II and above were less likely to be infected with HPV-18 compared with women who had FIGO cancer stage Ib when adjusted for ethnicity (adjusted odds ratio 0.5, 95% CI: 0.2–0.9; *P* = 0.029). The adjusted odds ratio for HPV-16 was not statistically significant (0.8, 95% CI: 0.6–1.4; *P* = 0.477).

### Co-infection with multiple HPV types

The prevalence of co-infection with multiple HPV types is shown in Table 3. The majority (93.5%, 188/201) of HPV positive women with ICC were infected with a single HPV type. Multiple HPV type infections were found in 5.5% of women, while in a further 1.0% of the HPV-positive samples the HPV type were unidentifiable by the typing test. Co-infections were more commonly found in SCC (6.1%) than in ADC/ASC (3.8%).

**Table 3:** **Co-infection of HPV types among HPV positive ICC tissue samples from New Zealand women (N**^**a**^ **= 201)**

**Histological diagnosis**	**HPV types**	**n**^**b**^	**%**^**c**^
**All ICC**			
N = 201	Any single infection	188	93.5
	Any multiple HPV types	11	5.5
	11/16	1	0.5
	16/18	1	0.5
	16/18/66	1	0.5
	16/31	1	0.5
	16/33	1	0.5
	16/45/52	1	0.5
	16/52	2	1.0
	18/35	1	0.5
	18/51	1	0.5
	18/59	1	0.5
	Unidentifiable HPV infection	2	1.0
**ICC subtypes**			
**ADC/ASC**	Any multiple HPV types	2	3.8
N = 53	16/18	1	1.9
	18/35	1	1.9
**SCC**	Any multiple HPV types	9	6.1
N = 148	11/16	1	0.7
	16/18/66	1	0.7
	16/31	1	0.7
	16/33	1	0.7
	16/45/52	1	0.7
	16/52	2	1.4
	18/51	1	0.7
	18/59	1	0.7

^a^number of women with the corresponding diagnosis.

^b^number of women in each category.

^c^percentage of women in each category.

## Discussion

This is the first study attempting to estimate the nationwide type distribution of HPV among NZ women diagnosed with ICC. Evidence of HPV infection was found in 88.5% of the histologically-confirmed ICC cases, and consistent with previous studies was higher in SCC than ADC/ASC cases. The majority (87.2%) were infected with HR HPV types. The distribution of HPV infection was highest in women aged 30–39 years. Since comprehensive data on the detection rate and type distribution of HPV among women with ICC in NZ are lacking, this study adds important information to this field.

Our findings on HPV detection rates are comparable with reports from the NZ region, Australia and international data. A single-region study (Auckland, NZ; 2000–2006) of 50 cervical biopsy specimens from women with confirmed ICC, reported 100% HPV infection and a 83.6% prevalence of HR HPV-16 and 18 [21]. Another study of HPV prevalence in liquid based cytology specimens of 594 patients from the NZ-NCSP referred for colposcopy with high grade cervical lesions between 2009 and 2011, reported a HR HPV prevalence of 86.7% [22]. An international study including 10 575 cases of ICC from around the world including Australia but not NZ, reported that 85.0% of the women with ICC were HPV positive [11]. A recent meta-analysis with Oceania data from nine studies found that HPV prevalence varied substantially by region among women with normal cytology but was consistent at about 90% for ICC across all regions [23].

The HPV type distribution results from our study show that HPV-16 and HPV-18 were the most common HR HPV types. This is consistent with previous literature from the region [22,24-26] and globally [11]. In addition, the frequently detected non-16/18 HR HPV types in our study were HPV-31, 45, 52, 59 and 33 which is consistent with existing international data [23,26], data from Australia [27] and with recent data from NZ in high grade cervical intraepithelial neoplasia [22]. It is interesting to note that in the cytology based study, HPV-52 was found in 21% of NZ women with CIN3 compared with 3% of women with ICC in our study [22]. These findings highlight the importance of future monitoring of HPV genotypes in women with cervical cancer and the screening population.

Epidemiological studies of HPV type distribution in SCC and ADC conducted in Oceania and world-wide report that the prevalence of HPV-16 is higher in SCC while HPV-18 is more predominantly seen in ADC/ASC cases [11,26,28]. We also observed a similar trend with HPV-16 being present in 56% of SCC cases compared with 39.7% in ADC/ASC cases, and HPV-18 present in 35.3% of ADC/ASC cases compared with only 14.5% of SCC cases. However, more recent studies indicate that HPV-16 and -18 constitute more than 80% of HPV types detected in women with cervical ADC [29,30].

Of particular interest, we found that HPV-16 was detected more frequently in women aged < 30 years than in other age groups, which is in accordance with previous studies [11,31-34]. The highest relative prevalence of type 16/18 versus other HR HPV types was also reported recently among women aged 20–29 years in a study among women with high grade cervical intraepithelial lesions [23]. We also found a lower proportion of HPV 16/18 or HPV 18 positive cases in advanced stage disease. There is little published information regarding the correlation of HPV type and cancer stage.

The reasons for these trends are unclear. More advanced disease is progressively common in older women. It is possible that 16/18 types are simply more prevalent in younger women, HPV may be less well preserved or expressed in more advanced tumours, or differences in the tumour biology may be important. One explanation may be that HPV types 16, 18 and 45 are more likely to be integrated into the human genome compared with other types and as a result have a more rapid natural history in episomal form, resulting in detection at a younger age or earlier stage of cancer. As younger women are better screened they may present at earlier stage of cervical abnormality [34]. Further investigation of these matters is warranted, as they may help inform the design of future screening (cervical smear or HPV DNA testing) or vaccination programs.

Ethnicity and its associations with HPV type distribution were assessed, although this was not the primary objective and the study was not sufficiently powered to test any hypothesis around this. It has been estimated that Maori women have an approximately 2-fold higher incidence of ICC [35]. For the years 1991–2004, Maori women with CC had a 61% greater excess mortality (poorer survival) compared with non-Maori women [8]. However, no differences in ICC subtype distribution and stage of cancer were observed between Maori and non-Maori women with ICC. Only minor variations in non HPV-16/18 types were observed between the Maori and non- Maori women. Our epidemiological findings do not explain the existence of the higher incidence or mortality burden in Maori women compared with non-Maori women. The slightly higher proportion of Maori women with HPV-16 infection in this study might be expected with the younger age, lower screening rate and higher SCC rates reported previously in this group.

An earlier study from Auckland, NZ, found that the prevalence of multiple HPV type infections among ICC is 14.2% [22] compared with the 5.5% reported in our study. This discrepancy may be explained by technological differences in the technique used for DNA extraction and HPV typing between studies, selection and size of tissue samples, complexity of the lesions, or real differences in the populations investigated. Multiple infections with different HPV types in the genital tract are not uncommon, with lower grade squamous lesions adjacent to the tumour often present in ICC tissue sections.

Our study has several strengths. It is the first national study reporting representative and recent data among women with ICC in NZ. The majority of the CC treatment in NZ is performed by or in conjunction with the public hospitals regularly treating ICC. Therefore, as CC treatment of women with FIGO stage Ib or greater is centralized to the participating hospitals in the study, the study design offered good representativeness of the population. Moreover, the cervical tissue samples were tested in a central laboratory providing a uniform diagnostic approach with standardized process of sample handling, HPV DNA detection and type identification using a highly sensitive assay.

The limitations of this study include the large number of screened women who were not included in the study, the lack of access to original tissues by the reviewing pathologists, variable quality of paraffin embedded tissue samples and self reporting of ethnicity which might have led to misclassification. Furthermore, since we did not include DNA controls to further test the HPV negative samples, some of the HPV negative samples might be false negatives. The detection rate in this study might therefore underestimate the true prevalence of HPV.

The study aimed to be representative of all women with ICC in New Zealand. Due to ethical requirements all women or their next of kin had to give informed consent for the samples to be included in the study and as a result only 242 of 402 screened women were enrolled. This has the potential to bias the study inclusion on socioeconomic, geographical, age ethnicity and disease status. While the age range of women included in this analysis is comparable with national cervical cancer registration figures, only 15% of women were Maori, slightly lower than in cancer registration figures and suggesting this group is slightly underrepresented here. While these matters must be remembered when drawing conclusions from this cross-sectional study the authors believe this is the most accurate possible assessment of HPV detection rate in NZ women with ICC prior to the introduction of the HPV vaccination program.

Patients with FIGO stage 1a were excluded as stage 1a tumours would be less likely to have available material due to serial sectioning of tumour for diagnostic reasons and referral to tertiary centres could not be guaranteed. The study is therefore not representative for this group. Finally, while cross-sectional study designs are widely accepted and utilised, the results only reflect a single time point estimation meaning HPV types cannot be linked to outcomes.

Despite these limitations, the study reports recent epidemiological data which can facilitate future policy and strategy decisions for HPV vaccination programmes and cervical screening in NZ.

Currently, the vaccination programme in NZ is poorly attended. Of the girls from the 1992 birth cohort (whose eligibility for free vaccination expired in 2012), only 51% completed the three-dose schedule for the quadrivalent HPV vaccine [12]. Australia, one of the first countries to introduce a national immunization program in 2007 has good coverage rates with 73% of school-aged girls having received all three doses of the vaccine [36].

Considering that women from poorer socioeconomic groups, Maori and other ethnicities have lower utilization of screening, more will need to be done to drive the uptake of screening as well as immunization in these groups to ensure equitable benefits and reduce the morbidity and mortality due to HPV.

## Conclusion

Findings from the study fill an important gap in epidemiological data on HPV type distribution in ICC patients from NZ which will help facilitate better understanding of the potential impact of HPV vaccines on the prevention of CC in NZ women, especially vaccines that provide broader protection beyond HPV-16/18.

### Trademarks

*Cervarix* is a trademark of the GlaxoSmithKline group of companies.

*Gardasil* is a trademark of Merck & Co. Inc.

*SAS* is a trademark of SAS Institute Inc.

## Abbreviations

ADC: Adenocarcinoma; ASC: Adenosquamous carcinoma; CC: Cervical cancer; CI: Confidence interval; DEIA: DNA enzyme immunoassay; FIGO: International federation of gynecology and obstetrics; HPV: Human papillomavirus; HR: High-risk; ICC: Invasive cervical cancer; LiPA: Line probe assay; LR: Low-risk; NCSP: National cervical screening programme; NZ: New Zealand; SCC: Squamous cell carcinoma; SD: Standard deviation; SPF-10: Short polymer fragment-10.

## Competing interest

JC and GK are employees of the GlaxoSmithKline group of companies. JC holds restricted shares and stock options from GSK. AM’s employer (DDL Diagnostic Laboratory) received funding from GSK for HPV testing of samples in this study. PS, DK, AT and SP declare no competing interest.

## Authors’ contributors

PS was the principal investigator, and together with AT, DK and SP were responsible for conducting the study at respective sites. PS, JC and KG contributed to the study design. KG performed the statistical analyses. AM was responsible for HPV genotyping. All authors contributed to the analysis and interpretation of results. All authors had access to the data and participated in the critical review of manuscript drafts and approval the final version.

## Pre-publication history

The pre-publication history for this paper can be accessed here:

http://www.biomedcentral.com/1471-2334/14/374/prepub
